# Role of Machine Learning Techniques to Tackle the COVID-19 Crisis: Systematic Review

**DOI:** 10.2196/23811

**Published:** 2021-01-11

**Authors:** Hafsa Bareen Syeda, Mahanazuddin Syed, Kevin Wayne Sexton, Shorabuddin Syed, Salma Begum, Farhanuddin Syed, Fred Prior, Feliciano Yu Jr

**Affiliations:** 1 Department of Neurology University of Arkansas for Medical Sciences Little Rock, AR United States; 2 Department of Biomedical Informatics University of Arkansas for Medical Sciences Little Rock, AR United States; 3 Department of Surgery University of Arkansas for Medical Sciences Little Rock, AR United States; 4 Department of Health Policy and Management University of Arkansas for Medical Sciences Little Rock, AR United States; 5 Department of Information Technology University of Arkansas for Medical Sciences Little Rock, AR United States; 6 College of Medicine Shadan Institute of Medical Sciences Hyderabad India; 7 Department of Radiology University of Arkansas for Medical Sciences Little Rock, AR United States

**Keywords:** COVID-19, coronavirus, SARS-CoV-2, artificial intelligence, machine learning, deep learning, systematic review, epidemiology, pandemic, neural network

## Abstract

**Background:**

SARS-CoV-2, the novel coronavirus responsible for COVID-19, has caused havoc worldwide, with patients presenting a spectrum of complications that have pushed health care experts to explore new technological solutions and treatment plans. Artificial Intelligence (AI)–based technologies have played a substantial role in solving complex problems, and several organizations have been swift to adopt and customize these technologies in response to the challenges posed by the COVID-19 pandemic.

**Objective:**

The objective of this study was to conduct a systematic review of the literature on the role of AI as a comprehensive and decisive technology to fight the COVID-19 crisis in the fields of epidemiology, diagnosis, and disease progression.

**Methods:**

A systematic search of PubMed, Web of Science, and CINAHL databases was performed according to PRISMA (Preferred Reporting Items for Systematic Reviews and Meta-Analysis) guidelines to identify all potentially relevant studies published and made available online between December 1, 2019, and June 27, 2020. The search syntax was built using keywords specific to COVID-19 and AI.

**Results:**

The search strategy resulted in 419 articles published and made available online during the aforementioned period. Of these, 130 publications were selected for further analyses. These publications were classified into 3 themes based on AI applications employed to combat the COVID-19 crisis: Computational Epidemiology, Early Detection and Diagnosis, and Disease Progression. Of the 130 studies, 71 (54.6%) focused on predicting the COVID-19 outbreak, the impact of containment policies, and potential drug discoveries, which were classified under the Computational Epidemiology theme. Next, 40 of 130 (30.8%) studies that applied AI techniques to detect COVID-19 by using patients’ radiological images or laboratory test results were classified under the Early Detection and Diagnosis theme. Finally, 19 of the 130 studies (14.6%) that focused on predicting disease progression, outcomes (ie, recovery and mortality), length of hospital stay, and number of days spent in the intensive care unit for patients with COVID-19 were classified under the Disease Progression theme.

**Conclusions:**

In this systematic review, we assembled studies in the current COVID-19 literature that utilized AI-based methods to provide insights into different COVID-19 themes. Our findings highlight important variables, data types, and available COVID-19 resources that can assist in facilitating clinical and translational research.

## Introduction

COVID-19 is a global health crisis, with more than 16 million people infected and over 666,000 deaths reported (up to July 29, 2020) worldwide [1]. The resulting impact on health care systems is that many countries have overstretched their resources to mitigate the spread of the pandemic [2]. In addition, a high degree of variance in COVID-19 symptoms has been reported, with symptoms ranging from a mild flu to acute respiratory distress syndrome (ARDS) or fulminant pneumonia [3-5]. There is an urgent need for effective drugs and vaccines for COVID-19 treatment and prevention. Owing to the lack of validated therapeutics, most containment measures to curtail the spread of the disease rely on social distancing, quarantine measures, and lockdown policies [2,6]. The transmission of COVID-19 has been slowed as a result of these measures, but not eliminated. Moreover, with the ease of restrictions, a fear of the second wave of infection is prevalent [7,8]. To prevent the second potential outbreak of COVID-19, there is a need for advanced containment measures such as contact tracing and identification of hotspots [9,10].

Artificial intelligence (AI) encompasses a broad spectrum of technologies that aim to imitate cognitive functions and intelligent behavior of humans [11]. Machine learning (ML) is a subfield of AI that focuses on algorithms that enable computers to define a model for complex relationships or patterns from empirical data without being explicitly programmed [11]. Deep learning (DL), a subcategory of ML, achieves great power and flexibility compared to conventional ML models by drawing inspiration from biological neural networks to solve a wide variety of complex tasks, including the classification of medical imaging and natural language processing (NLP) [11].

AI techniques have been employed in the health care domain on different scales ranging from the prediction of disease spread trajectory to the development of diagnostic and prognostic models [12-14]. A study by Ye et al [15] identified and evaluated various health technologies, such as big data, cloud computing, mobile health, and AI, to fight the pandemic. These technologies and a wide range of data types, including data from social media, radiological images, omics, drug databases, and public health agencies, have been used for disease prediction [1,14,16-19]. Several studies have focused on reviewing publications that discuss AI applications to support the COVID-19 response [12,13,15,20,21]. One of the early studies by Vaishya et al [20] identified 7 critical areas where AI can be applied to monitor and control the COVID-19 pandemic. However, given that this was an early work, this review lacked publications in all the 7 areas. In a later study, Lalmuanawma et al [12] built upon these 7 areas by identifying and performing a rapid review of the then available studies; however, considering this was a rapid review, only limited studies were included, and the qualification criteria were not clear. Furthermore, a study by Shi et al [21] focused on AI applications to radiological images, and a study by Wynants et al [13] focused on critical appraisal of models that aimed to predict the risk of developing the disease, hospital admission, and disease progression. Nevertheless, the majority of epidemiological studies that aimed to model disease transmission or fatality rate, among other factors, were excluded in this study.

The primary aim of this study was to conduct a comprehensive systematic literature review on the role of AI as a technology to combat the COVID-19 crisis and to assess its application in the epidemiological, clinical, and molecular advancements. Specifically, we summarized the areas of AI application, data types used, types of AI methods employed and their performance, scientific findings, and challenges experienced in adopting this technology.

## Methods

This systematic literature review followed the guidelines of PRISMA (Preferred Reporting Items for Systematic Reviews and Meta-Analyses) framework for preparation and reporting [22].

### Eligibility Criteria

This study focused on peer-reviewed publications as well as preprints that applied AI techniques to analyze and address the COVID-19 crisis on different scales, including diagnostics, prognostics, disease spread forecast, omics, and drug development.

### Data Sources and Search Strategy

**Figure 1:**
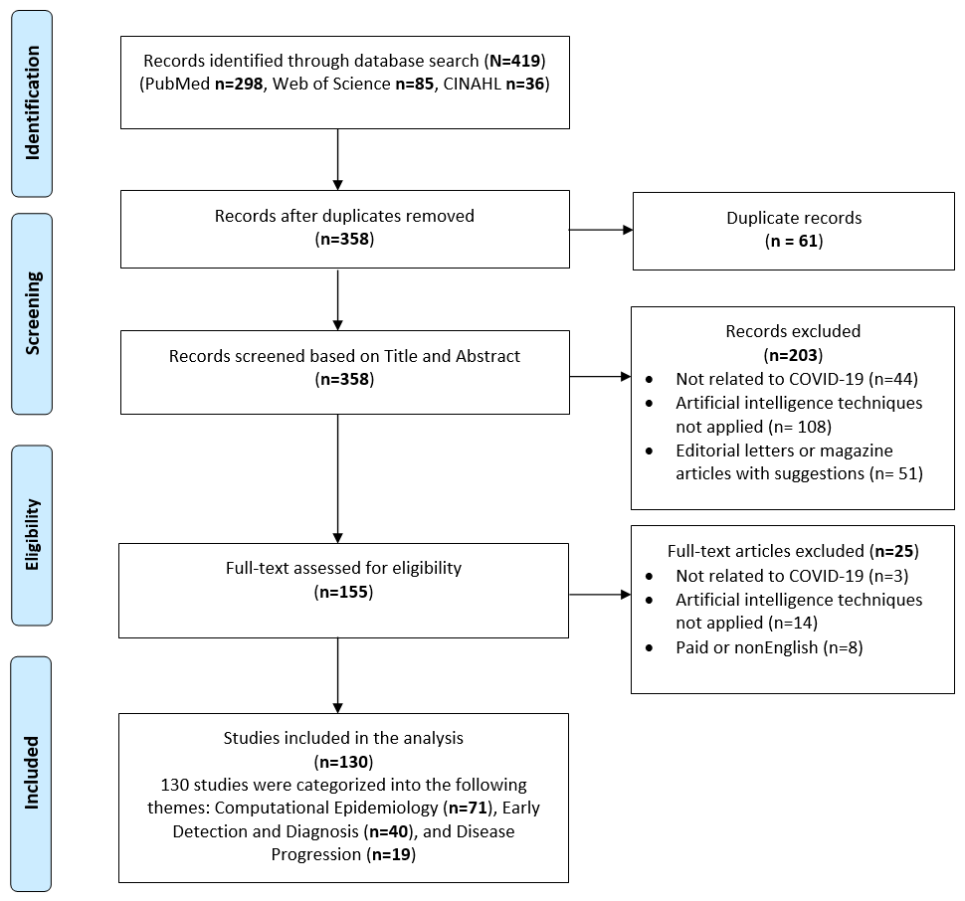
PRISMA (Preferred Reporting Items for Systematic Reviews and Meta-Analysis) flow diagram of systematic identification, screening, eligibility, and inclusion of publications that applied artificial intelligence techniques to tackle the COVID-19 pandemic.

PubMed, Web of Science, and CINAHL databases were searched, restricting the search to research articles published in English and in peer-reviewed or preprint journals or conference proceedings available from Dec 1, 2019, through June 27, 2020. The search syntax was built with the guidance of a professional librarian and included the following search terms: “CORONAVIRUS,” “COVID-19,” “covid19,” “cov-19,” “cov19,” “severe acute respiratory syndrome coronavirus 2,” “Wuhan coronavirus,” “Wuhan seafood market pneumonia virus,” “coronavirus disease 2019 virus,” “SARS-CoV-2,” “SARS2,” “SARS-2,” “2019-nCoV,” “2019 novel coronavirus,” “novel corona,” “Machine Learning,” “Artificial Intelligence,” “Deep Learning,” “Neural Network,” “Random Forest,” “Support Vector Machine,” and “SVM.” Refer to Multimedia Appendix 1 for search query syntax. Figure 1 illustrates the process of identifying eligible publications.

### Study Selection

Following the systematic search process, 419 publications were retrieved. Of that, 61 duplicate publications were removed, leaving 358 potentially relevant articles for title and abstract screening. Two teams of reviewers (HB, SS and MS, SB) screened these articles independently, following which an additional 203 publications were removed, and 155 publications were retained for a full-text assessment. These publications were further assessed for eligibility, resulting in a total of 130 publications that were included in the final analysis. Disagreements between reviewers were resolved by an independent review by a third reviewer (FS).

### Data Collection and Analyses

Qualitative and quantitative descriptive analyses were performed on the included studies (n=130) that had used AI techniques for tackling the COVID-19 pandemic. Based on the area of application, the studies were categorized into the following 3 themes: (1) Computational Epidemiology (CE), (2) Early Detection and Diagnosis (EDD), and (3) Disease Progression (DP). Qualitative analysis was performed on studies that belonged to the CE theme and quantitative descriptive analysis was performed for studies that belonged to the EDD and DP themes. After data extraction and analysis, we summarized and reported the findings in the form of tables and figures in accordance with the aim of the study.

## Results

### Search Results

The search strategy yielded a total of 419 articles, which were published and made available between December 1, 2019, and June 27, 2020. Of which, 130 publications were selected for further analyses. These 130 publications were categorized into 3 themes (ie, CE, EDD, and DP) based on the various AI applications employed to combat the COVID-19 crisis. These themes were identified based on AI techniques used to predict, classify, assess, track, and control the spread of the virus. Descriptions of each theme and related publications are presented in Table 1.

**Figure 2:**
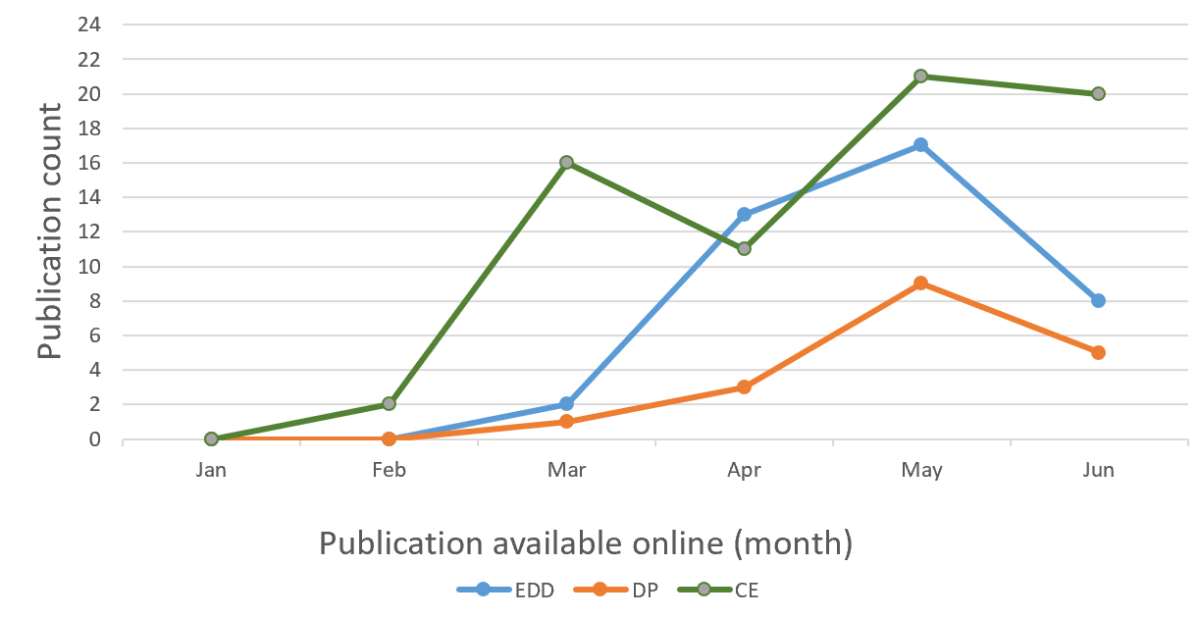
Over time (trend analysis) of COVID-19 studies focused on the application of artificial intelligence techniques that were made available online in 2020, categorized into the following 3 themes: (1) Computational Epidemiology (CE), (2) Early Detection and Diagnosis (EDD), and (3) Disease Progression (DP). For preprint articles, the publication month of the latest version available as of query search date was used.

During the initial days of the COVID-19 outbreak, the majority of published studies focused on predicting the outbreak and potential drug discoveries; we identified 71 such studies and classified them into the CE theme. Furthermore, 40 studies that applied AI techniques to detect COVID-19 using patients’ radiological images or laboratory test results were grouped under the EDD theme. Finally, 19 studies that focused on predicting disease progression, outcomes (recovery and mortality), length of stay, and the number of days spent in the intensive care unit (ICU) for patients with COVID-19 were grouped under the DP theme. Over time trend of COVID-19 publications by month and themes is shown in Figure 2, which depicts an initial surge of publications focusing on the CE theme followed by the EDD theme.

**Table 1:** An overview of the 130 publications in the literature, classified into 3 themes and their descriptions. The themes are listed according to the frequency of publication (percentage and absolute count).

Theme	Description	References	Publication count, n (%)
Computational Epidemiology	Publications focused on the development and application of artificial intelligence models to tackle issues central to epidemiology, such as disease trends and forecast of potential outbreak, pathobiology of coronavirus infection, protein structures, potential drug discoveries, policies, and social impact.	[14,16,18,23-90]	71 (54.6)
Early Detection and Diagnosis	Publications focused on the application of artificial intelligence techniques to detect and differentiate patients with COVID-19 from the general population.	[91-130]	40 (30.8)
Disease Progression	Publications focused on the application of artificial intelligence models to predict disease progression, severity, and likely outcomes in the confirmed COVID-19 population.	[17,131-148]	19 (14.6)

### Publications Focused on CE

The 71 studies that focused on epidemiological concerns of COVID-19 were further classified into 3 categories: (1) COVID-19 disease trajectory (CDT), (2) molecular analysis-drug discovery (MADD), and (3) facilitate COVID-19 response (FCR). These classifications were based on the study aims, that is, to predict outbreaks, potential drug discoveries, policies, and other measures to contain the spread of COVID-19 (see Table 2). In all, 40 studies that focused on predicting COVID-19 peaks and sizes globally and specific to a geographical location, estimating the impact of socioeconomic factors and environmental conditions on the spread of the disease, and effectiveness of social distancing policies in containing disease spread were categorized under CDT. Next, 22 publications were grouped under MADD based on the study approach used, including studies focused on identification of existing drugs that have the potential to treat COVID-19, analysis of protein structure, and prediction of mutation rate in patients with COVID-19. Finally, 9 studies that emphasized on building tools to combat the ongoing pandemic, such as building a COVID-19 imaging repository, AI-enabled automatic cleaning and sanitizing tasks at health care facilities that might assist clinical practitioners to provide timely services to the affected population, were categorized under FCR. The majority of publications classified under the CE theme used data from either social media (eg, 9 studies used data from Twitter, Weibo, or Facebook) or public data repositories (Eg, NCBI, DrugBank databases, and other health agencies). Details of individual studies are provided in Multimedia Appendix 2.

**Table 2:** Computational Epidemiology publications (n=71) subclassified into 3 categories: (1) COVID-19 disease trajectory, (2) molecular analysis-drug discovery, and (3) facilitate COVID-19 response.

Category	Description	References	Publication count, n (%)
COVID-19 disease trajectory	Publications focused on predicting COVID-19 peaks and sizes globally (and specific to a geographical location), estimating the impact of socioeconomic factors and environmental conditions on the spread of the disease, and effectiveness of social distancing policies in containing disease spread.	[23-62]	40 (56.3)
Molecular analysis: drug discovery	Publications focused on identifying existing drugs that have the potential to treat COVID-19, analysis of protein structure, and predicting mutation rate in patients with COVID-19.	[69-89]	22 (31)
Facilitate COVID-19 response	Publications focused on building tools to combat the ongoing pandemic, such as building a COVID-19 imaging repository, artificial intelligence–enabled automatic cleaning and sanitizing tasks at health care facilities to assist clinical practitioners to provide timely services to the affected population.	[63-68,90]	9 (12.7)

### Publications Focused on EDD

We identified 40 publications that primarily focused on diagnosing COVID-19 in patients with suspected infection mostly by using chest radiological images, such as computed tomography (CT), X-radiation (X-ray), and lung ultrasound (LUS). As shown in Table 3, 23 studies used X-ray, 15 used CT, 1 study used LUS, and 1 study used nonimaging clinical data. Most studies used DL techniques to diagnose COVID-19 based on radiological images. Nine studies employed ResNet, 4 studies used Xception, and 3 studies used VGG neural network models either for pretraining or as a diagnostic model. The only study that used nonimaging clinical data to diagnose COVID-19 employed routine laboratory results captured in electronic health record (EHR) systems. Details of individual studies are provided in Multimedia Appendix 3.

**Table 3:** Early Detection and Diagnosis publications (n=40) subclassified into 4 categories based on the modality used for COVID-19 prediction: (1) X-ray, (2) computed tomography, (3) lung ultrasound, and 4) nonimaging clinical data.

Category	Description	References	Publication count, n (%)
X-ray	Publications focused on the application of artificial intelligence techniques to detect and differentiate patients with COVID-19 from the general population using X-ray images.	[91, 92, 95, 97-99, 101-106, 112-114, 117, 118, 122-124, 126, 127, 129]	23 (57.5)
Computed tomography	Publications focused on the application of artificial intelligence techniques to detect and differentiate patients with COVID-19 from the general population using computed tomography images.	[93, 94,96, 100, 107-111, 116,119-121, 125, 130]	15 (37.5)
Lung ultrasound	Publication focused on the application of artificial intelligence techniques to detect and differentiate patients with COVID-19 from the general population using lung ultrasound images.	[128]	1 (2.5)
Nonimaging clinical data	Publication focused on the application of artificial intelligence techniques to detect and differentiate patients with COVID-19 from the general population using nonimaging clinical data.	[115]	1 (2.5)

### Publications Focused on DP

We identified 19 publications that were primarily focused on the prognosis of disease in patients with COVID-19. We further classified these studies into (1) risk stratification (n=15), which included publications focused on assessing the risk of DP and (2) hospital resource management (n=4), which included publications focused on predicting the need for hospital resources (see Table 4). All 15 DP studies used demographic variables, 13 studies used comorbidities, and 11 studies used radiological images for analyses. Details of individual studies grouped under this theme are provided in Multimedia Appendix 4.

**Table 4:** Disease Progression publications subclassified into 2 categories: (1) risk stratification and (2) hospital resource management.

Category	Description	References	Publication count, n (%)
Risk stratification	Publications focused on assessing the risk of disease progression.	[17, 131, 133, 134, 137-140, 142-148]	15 (78.9)
Hospital resource management	Publications focused on predicting the need for hospital resources.	[132, 135, 136, 141]	4 (21.1)

## Discussion

AI techniques will continue to be used for the monitoring, detection, and containment of the COVID-19 pandemic [56,95,131]. Our systematic review focused on 130 studies that applied AI methods and identified 3 broad themes: models developed to address issues central to epidemiology, models that aid the diagnosis of patients with COVID-19, and models that facilitate the prognosis of patients with COVID-19. The 7 areas of AI application areas as identified by Vaishya et al [20] were grouped into these themes, as described below.

### Theme 1: CE models

In this theme, we review various AI techniques applied in different areas of epidemiology.

#### AI Techniques for MADD

##### Current State of Drug Discovery for COVID-19

Currently, there is no available vaccine for treating COVID-19 patients, and this has forced researchers to invent new strategies for expediting antiviral treatment and decreasing the mortality rate [149]. On average, the conventional drug discovery process takes 10-15 years and has very low success rates [150]. Instead, drug repurposing attempts have been made to explore similarities between SARS-CoV-2 (ie, the causative agent of COVID-19) and other viruses such as SARS and HIV [151]. With the rapid accumulation of genetic and other biomedical data in recent years, AI techniques facilitate the analyses of drugs and chemical compounds that are already available to find new therapeutic indications [152].

##### Protein Structure Analysis

The main protease (Mpro) of COVID-19 is a key enzyme in polyprotein processing, which plays an important role in mediating viral replication and transcription [153]. Several studies have applied AI techniques to identify drug leads that target Mpro of SARS-CoV-2, thereby making it an attractive drug target [154,155]. Ton et al [87] built a DL platform called Deep Docking, which enables structure‐based virtual screening of billions of purchasable molecules in a short time. This platform was used to process more than 1 billion compounds available from the ZINC15 library in order to identify the top 1000 potential ligands for SARS‐CoV‐2 Mpro. The proposed docking platform is a computationally cheaper and faster AI method than traditional docking methods, which allows faster screening of large chemical libraries containing billions of compounds.

##### Drug Repurposing

Beck et al [16] used a drug-target interaction DL model to identify the top 10 commercially available drugs that could act on viral proteins of SARS-CoV-2. The DL model called Molecule Transformer-Drug Target Interaction was used to predict binding affinity values between marketable antiviral drugs that could target COVID-19 proteins. The researchers claim that this model can accurately predict binding affinity based on chemical and amino acid sequences of a target protein without knowledge of their structural information. Moreover, the study reports that Atazanavir is the most effective chemical compound with K_d_ of 94.94 nM, followed by Remdesivir (113.13 nM), Efavirenz (199.17 nM), Ritonavir (204.05 nM), and Dolutegravir (336.91 nM) against the SARS-CoV-2 3C-like proteinase. Computational drug repositioning AI models provide a fast and cost-effective way to identify promising repositioning opportunities, and expedited approval procedures [152,156].

##### Viral Genome Sequencing

Genome sequencing of various viruses is performed to identify regions of similarity that may have consequences for functional, structural, and evolutionary relationships [157]. Owing to the heavy computational requirements of traditional alignment-based methods, alignment-free genome comparison methods are gaining popularity [157,158]. A case study by Randhawa et al [84] proposed an ML-based alignment-free approach for an ultra-fast, inexpensive, and taxonomic classification of whole virus genomes for SARS-CoV-2 that can be used for classification of COVID-19 pathogens in real time.

#### AI Techniques for FCR

##### Ongoing FCR Initiatives

To combat the ongoing COVID-19 crisis, global scientific collaborations have been encouraged and are necessary now more than ever. Several initiatives are underway to build centralized repositories to share COVID-19–related research [159]. Such global repositories facilitate the understanding of disease characteristics, interventions, and potential mental health impacts on the general population.

##### Collaborative Open Source Repository

Peng et al [66] focused on creating a repository of COVID-19 chest X-ray (CXR) and chest CT images. The repository, COVID-19-CT-CXR, is publicly available and contains 1327 CT and 263 X-ray images (as of May 9, 2020) that are inadequately labeled. The authors build a pipeline to automatically extract images from the biomedical literature relevant to COVID-19 using a DL model. A recent effort by the National Center for Advancing Translational Sciences to build a centralized, national data repository on COVID-19, called National COVID Cohort Collaborative (N3C), is underway [160]. N3C will support collection and analyses of clinical, laboratory, and diagnostic data from hospitals and health care plans. N3C along with imaging repositories such as COVID-19-CT-CXR will accelerate clinical and translational research.

##### Psychological Impact of the COVID-19 Pandemic

COVID-19 lockdown and home-confinement restrictions have adverse effects on the mental well-being of the general population and specifically high-risk groups, including health care workers, children, and older adults [161]. Several studies have been conducted to understand and respond to these public health emergencies. For instance, Li et al [63] conducted a study using a ML model (support vector machine) and sentiment analysis to explore the effects of COVID-19 on people’s mental health and to assist policymakers in developing actionable policies that could aid clinical practitioners. Weibo posts were collected before and after the declaration of the pandemic to build emotional score and cognitive indicators. Key findings of the study reveal that after the declaration of the COVID-19 outbreak in China, there has been a significant impact resulting in increased negative emotions (eg, anxiety and depression) and sensitivity to social risks, and decreased happiness and satisfaction of life. Raamkumar et al [18] used the health belief model (HBM) [162] to determine public perception of physical distancing posts from multiple public health authorities. They used a DL (a variant of recurrent neural network) text classification model to classify Facebook comments related to physical distancing posts into 4 HBM constructs: perceived severity, perceived susceptibility, perceived barriers, and perceived benefits, with accuracy of the model ranging from 0.91 to 0.95. Moreover, recent developments in the field of NLP, bidirectional encoder representations from transformers [163], XLNet [164], and other hybrid ML models have shown promising results in the field of sentiment analysis. Future studies should focus on these advanced techniques for improved social media content analysis.

#### AI Techniques for CDT

##### Models for Prediction of COVID-19 Cases

During the initial days of the COVID-19 spread, most research was focused on building mathematical models for estimating the transmission dynamics and prediction of COVID-19 developments [165,166]. Specifically, susceptible-exposed-infectious-recovered (SEIR) and auto-regressive integrated moving average (ARIMA) models and their extensions were widely adopted for the projection of COVID-19 cases [167]. These models provided health care and government officials with optimal intervention strategies and control measures to combat the pandemic [167]. A similar suggestion was made by Lalmuanawma et al [12].

##### Forecasting of COVID-19

In our systematic review, Yang et al [59] and Moftakhar et al [44] used DL models to fit both statistical models SEIR and ARIMA. The long-short term memory model proposed by Yang et al [59] and artificial neural network model proposed by Moftakhar et al [44] had a good fit to SEIR and ARIMA, respectively. However, projections of both these mathematical models had deviations less than the ±15% range of the reported data [167]. Therefore, we recommend future studies should try to fit AI techniques on both the SEIR and ARIMA models to reduce the projection error rate and be better prepared for the second wave of COVID-19.

##### Impact of Policies on COVID-19 Trajectories

The accuracy of COVID-19 trajectory projections depends on varying containment policies enforced by different countries [167,168]. The study by Yang et al [59] used a DL technique to predict COVID-19 epidemic peaks and sizes with respect to the containment polices. Their study revealed that the continual enforcement of quarantine restrictions, early detection, and subsequent isolation were the most effective in containment of the disease. Relaxing these policies would likely increase the spread of disease by 3-fold for a 5-day delay in implementation and could cause a second peak. We suggest government officials should strictly enforce such policies to prevent a second outbreak of COVID-19.

### Theme 2: EDD models

#### Current State of COVID-19 Diagnosis

Many countries ramped up the production of real-time reverse transcription polymerase chain reaction (RT-PCR) testing kits to diagnose COVID-19, and thus far, it remains the gold standard for confirmed diagnosis [169]. However, this laboratory-based test is limited by low sensitivity, as reported by several studies [169,170]. As highlighted by both Vaishya et al [20] and Lalmuanawma et al [12], AI can prove helpful in the diagnosis of various infectious diseases (eg, SARS, HIV, and Ebola) when used in conjunction with medical imaging technologies such as CT, magnetic resonance imaging (MRI), and X-ray. Radiological images (CT and X-ray) have been used by clinicians to confirm COVID-19–positive cases; these imaging findings also serve as an important complement to the RT-PCR test [171]. In this systematic review, we found LUS has been used to diagnose COVID-19, in addition to CT and X-Ray. However, we did not find any study using MRI for COVID-19 diagnosis.

#### Diagnostic Models Based on CT and X-Ray

Several studies have reported that the use of chest CT for early-stage detection of COVID-19 has proven to have a low rate of misdiagnosis and can provide accurate results even in some asymptomatic cases [172]. We identified 15 studies that used CT to detect COVID-19. One of the most cited studies by Li et al [120] applied DL (COVNet) to differentiate COVID-19 and non–COVID-19 pneumonia CT scans. The area under the receiver operating characteristic (AUROC) curve reported to identify COVID-19 based on chest CT exam was 0.96 and the AUROC curve reported to identify community-acquired pneumonia based on chest CT exam was 0.95. The accuracy reported is slightly higher than that reported by Ardakani et al [93], which was also found in the review by Lalmuanawma et al [12]. However, there are some disadvantages associated with using chest CT for COVID-19 diagnosis, such as the high radiation dose (7 mSv vs 0.1 mSv for chest X-ray) and the fact that chest CT is more expensive than chest X-ray [173,174].

In this systematic review, we identified 23 studies that used chest X-ray and applied AI techniques to diagnose COVID-19 cases. A study by Apostolopoulos et al [91] applied a transfer learning strategy to train convolutional neural network models and then automated the detection of COVID-19 using chest X-ray images. The model (VGG19) achieved an overall accuracy of 97.82% to detect COVID-19 based on a dataset of 224 COVID-19, 700 pneumonia, and 504 normal X-ray images. A similar study was performed by Khan et al [117] using transfer learning and convolutional neural network (Xception) architecture with 71 layers that were trained on the ImageNet dataset. Their model (CoroNet) achieved an average accuracy of 87% to detect COVID-19 based on a dataset of 284 COVID-19, 657 pneumonia (both viral and bacterial), and 310 normal chest X-ray images. These recently published studies successfully used transfer learning strategy to overcome sample size limitation and adapt generalizability; it is noteworthy that such studies were not available in the earlier literature reviews [12,20]. Although chest X-ray is cost-effective and involves a considerably lower radiation dose than chest CT, it is less sensitive, especially in the early stages of the infection and in cases of mild disease [175]. We recommend that new studies develop AI models that can detect COVID-19 by using a combination of CT and X-ray images along with clinical variables to aid clinical practitioners with accurate diagnosis.

#### Diagnostic Models Based on LUS and Clinical Variables

During the 2009 influenza (H1N1) epidemic, LUS proved useful in accurately differentiating viral and bacterial pneumonia and were found to have higher sensitivity in detecting avian influenza (H7N9) than chest X-ray [176]. Although clinicians recommend the use of LUS imaging in the emergency room for the diagnosis and management of COVID-19, its role is still unclear [177]. In our review, we identified a study by Roy et al [128] that used a DL model based on an annotated LUS COVID-19 dataset to predict disease severity. The results of the study were reported to be “satisfactory.” Moreover, a study by Joshi et al [115] proposed an ML approach that utilizes only complete blood count and gender information of the patient to predict COVID-19 positivity, as an alternative to the RT-PCR test. These authors built a logistic regression model based on retrospective data collected from a single institute and validated using multi-institute data. Prediction of COVID-19 infection demonstrated a C-statistic of 78% and sensitivity of 93%. The aim of the study was to develop a decision support tool that integrates readily available laboratory test results from patients’ EHRs.

### Theme 3: DP Models

#### Current State of Predicting COVID-19 Progression

The COVID-19 pandemic has strained global health care systems, especially ICUs, due to the high ICU transfer rates of hospitalized patients with COVID-19 [135]. As the pandemic progressed, the research focus shifted from detecting the presence of the novel coronavirus in patient samples to the prediction of patient recovery and associated risks [178]. Therefore, early systematic reviews included very few studies that focused on DP [12,20]. In this review, we found 19 studies that predicted DP and the likely outcomes in the confirmed COVID-19 population. Prior identification of hospitalized patients who may be at high-risk may aid health care providers to more efficiently plan and prepare for ICU resources (eg, beds, ventilators, and staff) [179].

#### Hospital Resource Management

A study by Cheng et al [135] developed an ML-based model to predict ICU transfers within 24 hours of hospital admission. The random forest model was used for COVID-19 prediction and was based on multiple variables such as vital signs, nursing assessment, laboratory test results, and electrocardiograms collected during the patient’s hospital stay. The overall AUROC curve of the model was reported to be 79.9%. Similar work was done by Shashikumar et al [141] to predict the need for ventilation in hospitalized patients 24 hours in advance. The prediction was not only limited to patients with COVID-19 but also included other hospitalized patients. These authors studied 40 clinical variables, including 6 demographic and 34 dynamic variables (eg, laboratory results, vital signs, sequential organ failure assessment, comorbidity, and length of hospital stay). In contrast to the traditional ML model used by Cheng et al [135], Shashikumar et al [141] resorted to a DL model (VentNet) for prediction with an area under the curve (AUC) of 0.882 for the general ICU population and 0.918 for patients with COVID-19. Both the aforementioned studies relied on clinical variables for prediction, whereas a study by Burian et al [132] combined clinical and imaging parameters for estimating the need for ICU treatment. The major finding of the study was that the patients needing ICU transfers had significantly elevated interleukin-6, C-reactive protein, and leukocyte counts and significantly decreased lymphocyte counts. All studies in this category applied AI techniques to facilitate the efficient use of clinical resources and help hospitals plan their flow of operations to fight the ongoing pandemic.

#### Risk Stratification

Prediction and risk stratification of COVID-19 cases that are likely to have adverse outcomes will help to streamline health care resources for patients that need urgent care. In our review, Yadaw et al [146] evaluated different ML models to classify COVID-19 cases as deceased or alive classes. This classification was based on 5 features: age, minimum oxygen saturation during the encounter, type of patient encounter, hydroxychloroquine use, and maximum body temperature. Their study revealed that age and minimum oxygen saturation during encounters were the most predictive features among the different models examined. The overall AUC was reported as 0.91. On the other hand, Ji et al [137] focused on the early identification of COVID-19 cases that are likely to be at high-risk. Variables used for this prediction model included demographics, comorbidities, and laboratory test results. They found a strong correlation between comorbidities and DP as supported by various other studies. The study further suggests that a decrease in lymphocyte count and an increase in lactate dehydrogenase levels are related to DP. The overall AUC reported was 0.759. In both studies, Yadaw et al [146] and Ji et al [137], the ML models were trained on the retrospective data and validated on prospective data. Li et al [139] built a pulmonary disease severity score using X-rays and neural network models. The score was computed as the Euclidean distance between the patient’s image and a pool of normal images using the Siamese neural network. The score predicted (AUROC 0.80) subsequent intubation or death within 3 days of hospital admission for patients that were initially not intubated.

COVID-19 pneumonia is associated with high morbidity and mortality, and it is critical to differentiate COVID-19 from general pneumonia [180]. In the study by Jiang et al [138], their model used demographics, vital signs, comorbidities, and laboratory test results to predict patients that are likely to develop ARDS. Of these variables, laboratory levels of alanine aminotransferase (ALT), the presence of myalgias, and elevated hemoglobin were found to be the most predictive features. The overall accuracy of predicting ARDS was 80%. Moreover, using ALT alone, the model achieved an accuracy of 70%. Zhang et al [147] built a DL diagnostic and prognostic predictive model to detect COVID-19 and identified variables associated with risk factors for early intervention and monitoring of the disease. The study comprised 3777 patients (5468 CT scans) to differentiate COVID-19 pneumonia from other types of pneumonia and normal controls. The AUROC of the model was reported as 0.97.

### Distributed AI Architecture and Transfer Learning

The emergence of COVID-19 has encouraged public health agencies and scientific communities to share data and code, either by building data repositories or adopting federated AI models [13,181]. Moreover, transfer learning was adopted to fast-track AI model development, especially using imaging data.

#### Distributed AI Architecture

In general, DL techniques are employed to improve prediction accuracy by training models on large volumes of data [182]. In our review, several studies applied AI techniques, either using smaller imaging datasets specific to the organization, or mid- to large-sized datasets from publicly available repositories. However, there are substantial costs associated with the development and maintenance of such repositories [183]. To overcome data size and cost limitations, Xu et al [110] proposed a decentralized AI architecture to build a generalizable model that is distributed and trained on in-house client datasets, eliminating the need for sharing sensitive clinical data. The proposed framework is in the early phase of adoption and needs technical improvements before it is widely employed by participating health care organizations.

#### Transfer Learning

There are classification challenges associated with the diagnosis of COVID-19 using patients’ radiological imaging data, which consists of multiple steps. In general, the initial steps involved in image classification are preprocessing, annotation, and feature extraction. Annotation of radiological images is time-consuming and depends on the sheer expense of the expert radiologist. Several strategies have been proposed to address this challenge, such as self-supervised and transfer learning techniques. Our review identified a study by Wang et al [145] that used a transfer learning strategy to aid COVID-19 diagnostic and prognostic analyses. The study used a 2-step transfer learning strategy: first, the model was trained on a large lung cancer CT dataset (n=4106) along with epidermal growth factor receptor gene sequencing to learn associations between chest CT image and micro-level lung functional abnormalities. Thereafter, the model was trained and validated to differentiate COVID-19 from other pneumonia (AUC 0.87-0.88) and viral pneumonia (AUC 0.86) types. We believe such techniques will significantly improve the computational costs associated with training the models.

### Summary Points and Recommendations

The aim of this study was to perform a comprehensive literature review on the role of AI to tackle the current COVID-19 pandemic. The scope of our study was not restricted to a specific application, but to cover all possible areas used by AI-based approaches. The major findings from various COVID-19 studies and the recommendations for future research provided therein are enlisted below.

- RT-PCR remains the gold standard confirmatory test for COVID-19. However, this laboratory test has low sensitivity; therefore, future models should combine radiological images (eg, CT and X-ray), clinical manifestations, and laboratory test results for better accuracy.
- AI model performance might be biased due to lack of adequate sample size from small-scale studies. We suggest that newer studies should utilize data from national and international collaborative COVID-19 repositories. In addition, decentralized AI architecture should be adopted to eliminate the need for sharing sensitive clinical data.
- Most studies included at least some of the effective clinical variables for the prediction of COVID-19 progression. We have provided a comprehensive list of the variables used in the different studies and the best performing models reported therein. A detailed analysis of these variables should be performed to identify variables that are corelated with COVID-19 progression. Such variables should also be considered for future predictive models.
- Few studies have conducted a sentiment analysis using social media content and reported specific negative impacts on people’s mental health conditions due to the COVID-19 lockdown. Recent advancements in NLP techniques, such as transformers-based models and hybrid models, have been rarely used for sentiment analysis. We recommend that newer studies employ these advancements for improved analyses.
- Majority of the studies rarely provided details on how the AI model predictions were interpreted. Interpretable AI models allow end users to understand and improve model performance. Users can accept or decline the recommendations when such models are used as a clinical decision support tool.

### Limitations

This review has some inherent limitations. First, there is a possibility of studies missed due to the search methodology used. Second, we excluded 5 publications for which the full texts were not available, and this may have introduced bias. Third, we included studies that were available as preprints. Finally, a comparison of AI model performance was not possible in the quantitative descriptive analysis, as variables, sample size, and data sources varied across the selected studies. This systematic review includes publications that were available online as of June 27, 2020. As the COVID-19 pandemic progresses, we intend to perform another review on the studies published after the aforementioned date.

### Conclusions

In this systematic review, we assembled the current COVID-19 literature that utilized AI methods in the area of applications ranging from tracking, containing, and treating viral infection. Our study provides insights on the prospects of AI on the 3 identified COVID-19 themes—CE, EDD, and DP—highlighting the important variables, data types, and available COVID-19 resources that can assist in facilitating clinical and translational research. Our study sheds light on AI applications as a potential drug discovery and risk stratification tool. In addition, our analysis suggested that AI-based diagnostic tools are highly accurate in detecting the presence of the SARS-CoV-2 by using radiological imaging data and can be employed as a decision support tool.

## Appendix

Multimedia Appendix 1 Query syntax for study search in all 3 databases (PubMed, CINAHL, and Web of Science).

Multimedia Appendix 2 Details of 71 studies qualified under the Computational Epidemiology (CE) theme.

Multimedia Appendix 3 Details of 40 studies qualified under the Early Detection and Diagnosis (EDD) theme.

Multimedia Appendix 4 Details of 19 studies qualified under the Disease Progression (DP) theme.

## Abbreviations

AI: artificial intelligence
ALT: alanine aminotransferase
ARDS: acute respiratory distress syndrome
ARIMA: auto-regressive integrated moving average
AUC: area under the curve
AUROC: area under the receiver operating characteristics
CDT: COVID-19 disease trajectory
CE: computational epidemiology
CT: computed tomography
CXR: chest X-ray
DL: deep learning
DP: disease progression
EDD: early detection and diagnosis
EHR: electronic health record
FCR: facilitate COVID-19 response
HBM: health belief model
ICU: intensive care unit
LSTM: long-short term memory
LUS: lung ultrasound
MADD: molecular analysis-drug discovery
ML: machine learning
Mpro: main protease
MRI: magnetic resonance imaging
N3C: National COVID Cohort Collaborative
NLP: natural language processing
PRISMA: Preferred Reporting Items for Systematic Reviews and Meta-analysis
RT-PCR: reverse transcription–polymerase chain reaction
SEIR: specifically, susceptible-exposed-infectious-recovered
X-ray: X-radiation
