# Supplementary material for: Role of Machine Learning Techniques to Tackle the COVID-19 Crisis: Systematic Review
Source: JMIR Med Inform. 2021 Jan 11;9(1):e23811. doi: 10.2196/23811 (PMC7806275; doi:10.2196/23811)
Supplement: Multimedia Appendix 1 [file medinform_v9i1e23811_app1.docx]

Appendix 1: Query syntax for study search in all 3 databases. (PubMed, CINAHL, and Web of Science)

| **Database** | **Query Syntax** |
| --- | --- |
| PubMed | (  (CORONAVIRUS) OR (COVID-19) OR (covid19) OR (cov-19) OR (cov19)  OR (severe acute respiratory syndrome coronavirus 2) OR (Wuhan coronavirus)  OR (Wuhan seafood market pneumonia virus) OR (coronavirus disease 2019 virus)  OR (SARS-CoV-2) OR (SARS2) OR (SARS-2) OR (2019-nCoV) OR (2019 novel coronavirus)  OR (novel corona)  )  AND  (  (MACHINE LEARNING) OR (ARTIFICIAL INTELLIGENCE) OR (DEEP LEARNING) OR (NEURAL NETWORK) OR (Random Forest) OR (SVM) OR (Support Vector Machine)  ) |
| CINAHL | (  (CORONAVIRUS) OR (COVID-19) OR (covid19) OR (cov-19) OR (cov19)  OR (severe acute respiratory syndrome coronavirus 2) OR (Wuhan coronavirus)  OR (Wuhan seafood market pneumonia virus) OR (coronavirus disease 2019 virus)  OR (SARS-CoV-2) OR (SARS2) OR (SARS-2) OR (2019-nCoV) OR (2019 novel coronavirus)  OR (novel corona)  )  AND  (  (MACHINE LEARNING) OR (ARTIFICIAL INTELLIGENCE) OR (DEEP LEARNING) OR (NEURAL NETWORK) OR (Random Forest) OR (SVM) OR (Support Vector Machine)  ) |
| Web of Science | (  (CORONAVIRUS) OR (COVID-19) OR (covid19) OR (cov-19) OR (cov19)  OR (severe acute respiratory syndrome coronavirus 2) OR (Wuhan coronavirus)  OR (Wuhan seafood market pneumonia virus) OR (coronavirus disease 2019 virus)  OR (SARS-CoV-2) OR (SARS2) OR (SARS-2) OR (2019-nCoV) OR (2019 novel coronavirus)  OR (novel corona)  )  AND  (  (MACHINE LEARNING) OR (ARTIFICIAL INTELLIGENCE) OR (DEEP LEARNING) OR (NEURAL NETWORK) OR (Random Forest) OR (SVM) OR (Support Vector Machine)  ) |
