# Supplementary material for: Role of Machine Learning Techniques to Tackle the COVID-19 Crisis: Systematic Review
Source: JMIR Med Inform. 2021 Jan 11;9(1):e23811. doi: 10.2196/23811 (PMC7806275; doi:10.2196/23811)
Supplement: Multimedia Appendix 2 [file medinform_v9i1e23811_app2.docx]

Appendix 2: Details of 71 studies qualified under the Computational Epidemiology (CE) theme.

| **Study** | **Aim** | **Category** | **Source of data** |
| --- | --- | --- | --- |
| Arora et al, (2020) [23] | To predict COVID-19 positive patients in 32 states and union territories of India using a DL method. | COVID-19 Disease Trajectory | Ministry of Health and Family Welfare (Government of India). |
| Ayyoubzadeh et al, (2020) [24] | Study proposes the use of prediction models for COVID-19 incidence in Iran using Google Trends data. | COVID-19 Disease Trajectory | Google Trends data. |
| Car et al, (2020) [25] | Modeling the spread of COVID-19 infection using multilayer perceptron and time-series data of the infected patients. | COVID-19 Disease Trajectory | Johns Hopkins University Center for Systems Science and Engineering (JHU CSSE), ESRI Living Atlas Team, the Johns Hopkins University Applied Physics Lab. |
| Carrillo-Larco et al, (2020) [26] | To cluster countries that share similar COVID-19 profiles using an unsupervised ML model. | COVID-19 Disease Trajectory | Multiple sources including COVID-19 global surveillance system by Johns Hopkins University. |
| Chatterjee et al, (2020) [27] | Correlating the risk factors with COVID-19 and forecasting potential new cases and resulting deaths due to the pandemic. | COVID-19 Disease Trajectory | Two types of datasets.  a) Real datasets available in “ourworldindata.org” for time series forecasting and data visualization.  b) Simulated dataset. |
| Chimmula et al, (2020) [28] | Study evaluated the key features to predict the trends and possible stopping time of the current COVID-19 outbreak in Canada and around the world. | COVID-19 Disease Trajectory | Johns Hopkins University and Canadian Health authority. |
| Chowdhury et al, (2020) [29] | To build a multi-variable prediction model that allows to simulate COVID-19 outbreak trends in 16 countries. Then in each countries predict hospital usage, and mortality rates based on  1) no interventions done.  2) Consecutive iterations of mitigation measures followed by a relaxation period.  3) Consecutive cycles of suppression measures followed by a relaxation period. | COVID-19 Disease Trajectory | Data collected from 16 countries (Type of data includes Regional information, Economic status etc.). |
| Cobb et al, (2020) [30] | To predict COVID-19 growth rate trends among U.S counties with respect to stay at home policy using ML algorithm. | COVID-19 Disease Trajectory | Multiple sources including County metrics were obtained from the US Census Bureau, USA Counties (2011) data sets from the 2010 census. |
| Delen et al, (2020) [31] | Integrate multiple transactional data sets to study the role of social distancing policies in 26 countries and to analyze the transmission rate of the coronavirus disease (COVID-19) pandemic over the course of 5 weeks. | COVID-19 Disease Trajectory | Data collected from Google and Apple platform. |
| Fong et al, (2020) [32] | To Apply polynomial neural network with corrective feedback to accurately predict COVID-19 outbreak using small dataset. | COVID-19 Disease Trajectory | Empirical data collected from the archive of Chinese health authorities. |
| Fong et al, (2020) [33] | To gain better stochastic insights into COVID-19 development using Composite Monte-Carlo (CMC) simulation method enhanced by deep learning. | COVID-19 Disease Trajectory | Deterministic data from the Chinese Center for Disease Control and Prevention. Non-deterministic data. |
| Fronza et al, (2020) [34] | To support the design of containment policies using artificial neural network. | COVID-19 Disease Trajectory | Atmospheric data and Epidemiological data. |
| Golder et al, (2020) [35] | To identify the spread of COVID-19 using AI and data mining social networking sites in England (UK). | COVID-19 Disease Trajectory | Social media data collected from Twitter Streaming API. |
| Haghshenas et al, (2020) [36] | Use ML to prioritize and analyze the role of certain environmental parameters that effects spread of COVID-19 in Italy. | COVID-19 Disease Trajectory | Region specific environmental data from Italy. |
| Ismail et al, (2020) [37] | 14 days COVID-19 prediction/forecast using various ML models. | COVID-19 Disease Trajectory | European Center for Disease Prevention and Control. |
| Klein et al, (2020) [38] | To identify the spread of COVID-19 using AI and data mining techniques with social networking data. | COVID-19 Disease Trajectory | Social media data collected from Twitter Streaming API. |
| Liu et al, (2020) [39] | Forecast of COVID-19 activity (2-day ahead) using a novel method. | COVID-19 Disease Trajectory | COVID-19 activity reports from China Center for Disease Control and Prevention (CDC), Internet search activity from Baidu, news reports, and COVID-19 daily forecasts. |
| Liu et al, (2020) [40] | To model the trend of COVID-19 and estimate the restoration of operational capability of metropolitan medical service in China. | COVID-19 Disease Trajectory | Multiple sources including time series data collected from website of Tencent and population visualization by Baidu migration. |
| Mackey et al, (2020) [41] | To detect and characterize user-generated conversations that could be associated with COVID-19 symptoms, recoveries, and experiences with access to testing. | COVID-19 Disease Trajectory | Social media data collected from Twitter Streaming API. |
| Melin et al, (2020) [42] | Use of unsupervised self-organizing maps for grouping together similar countries in their fight against the coronavirus pandemic, and thus proposing these strategies for similar countries. | COVID-19 Disease Trajectory | Multiple sources including Humanitarian Data Exchange, dataset from the Mexico's Government website. |
| Melin et al, (2020) [43] | Predict COVID-19 using a multiple ensemble neural network model with fuzzy response aggregation. | COVID-19 Disease Trajectory | Mexico COVID-19 data (other sources not clear). |
| Moftakhar et al, (2020) [44] | To predict/forecast the number of daily new infected cases with COVID-19 for next thirty days in Iran. | COVID-19 Disease Trajectory | Data was prepared from daily reports of Iran Ministry of Health and open datasets provided by the John Hopkins University. |
| Mollalo et al, (2020) [45] | To predict the cumulative COVID-19 incidence rates across the United States. | COVID-19 Disease Trajectory | USAFacts, University of Washington Global Health Data Exchange. |
| Pirouz et al, (2020) [46] | To do analysis and predict COVID-19 cases. | COVID-19 Disease Trajectory | Environmental data (average temp, humidity, wind etc.). |
| Pourghasemi et al, (2020) [47] | Investigate spatial modeling, risk mapping, change detection, and outbreak trend analysis of the COVID-19 pandemic using ML. | COVID-19 Disease Trajectory | Iranian’s Ministry of Health and Medical Education, Worldometer, WorldClim, and Open Street Map. |
| Qiu et al, (2020) [48] | ML model to examine the role of various socioeconomic factors, including exogenous weather characteristics, in mediating the local and cross-city transmissions of the novel coronavirus 2019 (COVID-19) in China. | COVID-19 Disease Trajectory | Data collected from 32 provincial Health Commissions in China. |
| Rao et al, (2020) [49] | To estimate the growth rate increases of COVID-19 disease for each county in the U.S. Also, relate the findings to contextual factors. | COVID-19 Disease Trajectory | Multiple sources including Johns Hopkins University, NOAA, US Census Bureau, USDA, Kaiser Family Foundation. |
| Ribeiro et al, (2020) [50] | Study aimed at Short-term forecasting of COVID-19 cases in Brazil (one, three, and six-days). | COVID-19 Disease Trajectory | Data collected from Brazilian State Health Offices. |
| Saba et al, (2020) [51] | To model and forecast the prevalence of COVID-19 epidemic in Egypt using statistical and AI methods. | COVID-19 Disease Trajectory | Data collected by Egyptian Ministry of Health and Population. |
| Shen et al, (2020) [52] | To predict COVID-19 case counts in mainland China. | COVID-19 Disease Trajectory | Social media data collected from Weibo. |
| Simsek et al, (2020) [53] | Artificial Intelligence (AI)-driven mobilization strategy for mobile assessment agents for epidemics/pandemics. | COVID-19 Disease Trajectory | Crowdsensim simulator data. |
| Sujath et al, (2020) [54] | ML model to forecast COVID-19 spread in India. | COVID-19 Disease Trajectory | Kaggle COVID-19 dataset. |
| Tiwari et al, (2020) [55] | To predict COVID-19 outbreak in India using ML technique based on the disease pattern of China. | COVID-19 Disease Trajectory | Kaggle COVID-19 dataset provided by Center for Systems Science and Engineering (CSSE) at Johns Hopkins University (JHU). |
| Tomar et al, (2020) [56] | To forecast COVID-19 cases in India 30 days ahead. The effect of preventive measures like social isolation and lockdown on the spread of COVID-19. | COVID-19 Disease Trajectory | Data collected from Government of India COVID-19 Dashboard (National Portal of India). |
| Vaid et al, (2020) [57] | To forecast the occurrence of future COVID-19 related cases across relaxed (Sweden) and stringent (USA and Canada) policies. | COVID-19 Disease Trajectory | Oxford COVID-19 Government Response Tracker, New York Times data repository and data from other literature. |
| Wen et al, (2020) [58] | To perform a sentinel syndromic surveillance for COVID-19 and other influenza-like illnesses using a deep learning based approach. | COVID-19 Disease Trajectory | Mayo clinic data. |
| Yang et al, (2020) [59] | To predict the epidemic progression using SEIR epidemiological model. | COVID-19 Disease Trajectory | Epidemiological data based on daily COVID-19 outbreak reported by the National Health Commission of China. |
| Zheng et al, (2020) [60] | Build a hybrid AI model for COVID-19 prediction. | COVID-19 Disease Trajectory | Multiple sources including center of disease control of China data and News reports. |
| Zhou et al, (2020) [61] | A retrospective modelling analysis of the transmission dynamics in China and to evaluate the effectiveness of the strict government control strategies. | COVID-19 Disease Trajectory | Multiple sources including Tencent social media, National Health Commission of China, WHO. |
| Zhu et al, (2020) [62] | To predict the epidemic trend of COVID-19 in different countries and cities. | COVID-19 Disease Trajectory | Huami device sensor data, CDC data from different countries. |
| Han et al  (2020) [90] | Explore public opinion in the early stages of COVID-19 in China by analyzing Sina-Weibo texts in terms of space, time, and content. | Facilitate COVID-19 Response (FCR) | Social media data collected from Weibo. |
| Li et al, (2020) [63] | To explore the impacts of COVID-19 on people’s mental health and to assist policy makers to develop actionable policies, and help clinical practitioners (e.g., social workers, psychiatrists, and psychologists) provide timely services to affected populations. | Facilitate COVID-19 Response (FCR) | Social media data collected from Weibo. |
| Mahmoud et al, (2020) [64] | ML technique for concealed face detection for surveillance during COVID-19. | Facilitate COVID-19 Response (FCR) | Data taken from other published studies. |
| Obeid et al, (2020) [65] | Using ML model to prioritize the testing of coronavirus disease using unstructured patient data collected through telehealth visits. | Facilitate COVID-19 Response (FCR) | Unstructured telehealth visit documents collected from Medical University of South Carolina Health care. |
| Peng et al, (2020) [66] | To build a public database of COVID-19 CXR and CT images, "COVID-19-CT-CXR", that automatically extracts COVID-19 studies. ML model is used to classify images from CXR and CT using PubMed. | Facilitate COVID-19 Response (FCR) | Data collected from PubMed Central. |
| Raamkumar et al, (2020) [18] | To develop and evaluate deep learning model and to determine public perceptions towards physical distancing using Health Belief Model (HBM). | Facilitate COVID-19 Response (FCR) | Facebook comments posted by the public in response to the COVID-19 posts of three PHAs: Ministry of Health of Singapore (MOH), Centers for Disease Control and Prevention (CDC) and Public Health England (PHE). |
| Ramalingam et al, (2020) [67] | Use AI-enabled framework for automating the cleaning tasks through a Human Support Robot (HSR). | Facilitate COVID-19 Response (FCR) | Bing Image search. |
| Sear et al, (2020) [14] | Apply machine learning on social media to quantify COVID-19 content into anti and pro-vaccination community. | Facilitate COVID-19 Response (FCR) | Social media data including Facebook pages supporting anti and pro vaccine views. |
| Wahbeh et al, (2020) [68] | To identify topics, opinions, and recommendations about the COVID-19 pandemic discussed by medical professionals on the Twitter. | Facilitate COVID-19 Response (FCR) | Social media data collected from Twitter Streaming API. |
| Abdelmageed et al, (2020) [69] | To design a peptide-based vaccine to predict epitopes from the corona envelope (E) protein using immune informatics analysis. | Molecular Analysis-Drug Discovery | Multiple Sources including NCBI, GenBank. |
| Beck et al, (2020) [16] | Aim of the study was to use pre-trained deep learning-based drug-target interaction model called Molecule Transformer-Drug Target Interaction (MT-DTI) to identify commercially available drugs that could act on viral proteins of SARS-CoV-2. | Molecular Analysis-Drug Discovery | Multiple sources including NCBI database, Drug Target Common database. |
| Demirci et al, (2020) [70] | To perform a ML based micro RNA prediction analysis for the SARS-CoV-2 genome as a potential for anti-viral defense for COVID-19. | Molecular Analysis-Drug Discovery | Multiple sources including NCBI, GenBank, Mendeley Data, miRBase. |
| Gao et al, (2020) [71] | To identify potential 2019-nCoV drugs using ML that will allow to treat COVID-19. | Molecular Analysis-Drug Discovery | Multiple sources including PDBBind database, ChEMBL. Refer paper for details. |
| Gao et al, (2020) [72] | Develop and validate ML models with relatively low root-mean-square error to screen 1553 FDA-approved drugs and 7012 investigational or off-market drugs in DrugBank for SARS-CoV-2 or SARS-CoV 3CL (main) protease inhibitors. | Molecular Analysis-Drug Discovery | Multiple public databases such as ChEMBL, PDBbind, Drug Bank database. |
| Gussow et al, (2020) [73] | Using integrated comparative genomics and ML techniques identify key genomic features that differentiate SARS-CoV-2 and the viruses behind the two previous deadly coronavirus outbreaks, SARS-CoV and Middle East respiratory syndrome coronavirus (MERS-CoV), from less pathogenic coronaviruses. | Molecular Analysis-Drug Discovery | Data collected from NCBI. |
| Heo et al, (2020) [74] | Predicting and refining protein structure model of Severe Acute Respiratory Syndrome Coronavirus 2(SARS-CoV-2). | Molecular Analysis-Drug Discovery | Multiple sources including NCBI. |
| Ke et al, (2020) [75] | Using AI model to identify the approved drugs that has potential to treat COVID-19. | Molecular Analysis-Drug Discovery | Multiple sources including Drug Bank. |
| Kim et al, (2020) [76] | Identify potential therapeutics for COVID-19 using ML. | Molecular Analysis-Drug Discovery | Multiple sources including ChEMBL, GEO, LINCS drug perturbation database. |
| Liu et al, (2020) [77] | To evaluate and optimize peptide vaccine formulations for SARS-CoV-3 using a combinatorial ML method. | Molecular Analysis-Drug Discovery | Multiple data sources including NCBI and from other literature. |
| Mick et al, (2020) [78] | Using ML model to build 26-, 10- and 3-gene classifiers that differentiates COVID-19 from other acute respiratory illnesses. | Molecular Analysis-Drug Discovery | Multiple sources including data from University of California, San Francisco (UCSF) and Zuckerberg San Francisco General Hospital. |
| Mirabelli et al, (2020) [79] | Build a quantitative high-throughput screen to identify efficacious single agents and combination therapies against SARS-CoV-2. AI-based machine learning used to classify features of cells for infection and stress. | Molecular Analysis-Drug Discovery | Drug Bank database and some internal data. |
| Nguyen et al, (2020) [80] | To use ML and structural-based drug repositioning model to test the efficacy of existing FDA approved drugs to treat COVID-19. | Molecular Analysis-Drug Discovery | Multiple sources including PDBBind database, Drug Bank, and ChEMBL. |
| Ong et al, (2020) [81] | To predict COVID-19 protein candidates for the development of vaccine using ML models. | Molecular Analysis-Drug Discovery | Multiple Sources including NCBI and Uniprot. |
| Pathan et al, (2020) [82] | To predict the future rate of mutation for COVID-19 positive patients using RNN-based LSTM model. | Molecular Analysis-Drug Discovery | Data collected from NCBI GenBank database. |
| Qiang et al, (2020) [83] | Using ML model to predict COVID-19 infection risk. | Molecular Analysis-Drug Discovery | Data collected from Chinese National Genomics Data Center. |
| Randhawa et al, (2020) [84] | Aim to identify an intrinsic COVID-19 virus genomic signature using machine learning for an ultra-fast, scalable, and highly accurate classification of whole COVID-19 virus genomes. | Molecular Analysis-Drug Discovery | Multiple sources including NCBI database, Virus-Host, GenBank. |
| Song et al, (2020) [85] | To identify high-binding-affinity aptamers targeting SARS-CoV-2 RBD, using an ACE2 competition-based aptamers selection strategy and a ML screening algorithm. | Molecular Analysis-Drug Discovery | Multiple sources including RCSB PDB Data Bank, mfold web server. |
| Tang et al, (2020) [86] | Study aims to use AI to identify potential 47 lead compounds that can target SARS-CoV-2 key enzyme. | Molecular Analysis-Drug Discovery | Data collected from NCBI. |
| Ton et al, (2020) [87] | Apply novel deep learning platform – Deep Docking (DD) to 1.3 billion compounds from ZINC15 library to identify top 1,000 potential ligands for SARS‐CoV‐2 Mpro protein. | Molecular Analysis-Drug Discovery | Multiple sources including NCBI and from other studies. |
| Wu et al, (2020) [88] | To apply computational models of human RNA transcript localization to better understand the subcellular localization of the SARS-CoV-2 genome and its constituent sgRNAs. | Molecular Analysis-Drug Discovery | GenBank and other previous studies data. |
| Zhang et al, (2020) [89] | Using ML model identify potential drugs for 2019-nCoV 3C-like protease by performing drug screening against four chemical compound databases and a database of tripeptides. | Molecular Analysis-Drug Discovery | Multiple sources including Genome and Drug bank. |

List of Abbreviation used in Multimedia Appendix 2

| **Abbreviation** | **Description** |
| --- | --- |
| ACE2 | Angiotensin-Converting Enzyme II |
| AI | Artificial Intelligence |
| API | Application Programming Interface |
| CDC | Centers for Disease Control and Prevention |
| ChEMBL | Chemical European Molecular Biology Laboratory |
| CT | Computed Tomography |
| CXR | Chest X-Rays |
| DL | Deep Learning |
| ESRI | Environmental Systems Research Institute |
| FDA | Food and Drug Administration |
| GEO | Gene Expression Omnibus |
| LINCS | Library of Integrated Network-based Cellular Signatures |
| LSTM | Long short-term memory |
| MERS | Middle East Respiratory Syndrome |
| ML | Machine Learning |
| NCBI | National Center for Biotechnology Information |
| NOAA | National Oceanic and Atmospheric Administration |
| PDB | The Protein Data Bank |
| PDBbind | Protein Data Bank bind |
| PHA | Public Health Agency |
| RBD | Receptor-Binding Domain |
| RCSB | Research Collaboratory for Structural Bioinformatics |
| RNA | ribonucleic acid |
| SARS-Cov | Severe acute respiratory syndrome coronavirus |
| SEIR | Susceptible, Exposed (infected, but not yet infectious), Infectious (now can infect others), Removed |
| sgRNA | single guide RNA |
| U.S | United States |
| UK | United Kingdom |
| US | United States |
| USA | United States of America |
| USDA | United States Department of Agriculture |
| WHO | World Health Organization |
