# Supplementary material for: Role of Machine Learning Techniques to Tackle the COVID-19 Crisis: Systematic Review
Source: JMIR Med Inform. 2021 Jan 11;9(1):e23811. doi: 10.2196/23811 (PMC7806275; doi:10.2196/23811)
Supplement: Multimedia Appendix 3 [file medinform_v9i1e23811_app3.docx]

Appendix 3: Details of 40 studies qualified under the Early Detection and Diagnosis (EDD) theme.

| **Study** | **Aim** | **Modality** | **Sample Size** | **Model** | **Model Performance** |
| --- | --- | --- | --- | --- | --- |
| Apostolopoulos et al, (2020) [91] | To detect coronavirus from X-Ray of patients with common pneumonia, COVID-19 and normal incidents. | X-Ray | 1427 X-Ray images; 224 images with confirmed COVID-19, 700 images with confirmed common pneumonia, and 504 images of normal condition | Convolutional Neural Network with transfer learning. | Overall accuracy of detection 97.82% |
| Apostolopoulos et al, (2020) [92] | To automatically detect and classify pulmonary diseases based on X-Ray images. | X-Ray | 3905 X-Ray images | Convolutional Neural Network called MobileNet v2 | Accuracy 99.18% in detection of COVID-19 |
| Ardakani et al, (2020) [93] | To distinguish infection of COVID-19 from non-COVID-19 groups using CT. | CT | 1020 CT slices from 108 patients with laboratory proven COVID-19; 6 patients with other atypical and viral pneumonia diseases | ResNet-101 and Xception were best performed models | ResNet-101 AUC 0.994 Xception AUC 0.994 |
| Bai et al, (2020) [94] | To establish and evaluate an artificial intelligence (AI) system in differentiating COVID-19 and other pneumonia on chest CT and assess radiologist performance without and with AI assistance. | CT | 521 confirmed COVID-19 CT; 665 patients with non-COVID-19 | EfficientNet B4 deep neural network architecture for lung segmentation, followed by two-layer fully connected Neural Network to pool slices together (Final model) | Accuracy of final model 96% in distinguishing COVID-19 from non-COVID-19 pneumonia on chest CT |
| Benbrahim et al, (2020) [95] | To classify COVID-19 in chest X-Ray images collected from Kaggle repository. | X-Ray | 320 images classified into two categories: 160 images for patients affected by COVID-19 and 160 Normal images | Convolutional Neural Network (CNN) based models InceptionV3 and ResNet50 with Apache Spark framework | COVID-19 detection accuracy of 99.01% by the pre-trained InceptionV3 model and 98.03% for the ResNet50 model |
| Chaganti et al, (2020) [96] | To present a method that automatically segments and quantifies abnormal CT patterns commonly present in coronavirus disease 2019 (COVID-19), namely ground glass opacities and consolidations. | CT | 9749 chest CT volumes | Neural net (deep reinforcement learning followed by DI2IN; DenseUNet) | Pearson correlation coefficient between method prediction and ground truth for COVID-19 positive scans was calculated as 0.92 for PO (P < .001), 0.97 for PHO(P < .001), 0.91 for LSS (P < .001), 0.9 for LHOS (P < .001) |
| Das et al, (2020) [97] | To screen COVID-19 positive Chest X-Ray from other non-COVID-19 and/or healthy cases using deep learning. | X-Ray | Six different combinations of datasets (D1 to D6) are used. Refer to paper for details | Truncated Inception Net (Convolutional Neural Network) | Accuracy of 99.96% in classifying COVID-19 positive cases from combined pneumonia and healthy cases |
| El Asnaoui et al, (2020) [98] | To detect and classify coronavirus pneumonia from X-Ray images using deep learning method. | X-Ray | 6087 images (2780 images of bacterial pneumonia, 1493 of coronavirus, 231 of COVID-19, and 1583 normal) | Multiple Models, best performer Inception-ResNetV2 | Classification of coronavirus pneumonia ccuracy with Inception-ResNetV2 and Densnet201 reported to be 92.18% and 88.09% respectively |
| Elaziz et al, (2020) [113] | To classify the chest X-Ray images into two classes, COVID-19 patient or non-COVID-19 person using machine learning method. | X-Ray | First Data Set: contains 216 COVID-19 positive images (some collected from the Twitter account of Italian Cardiothoracic radiologist), 1,675 negative COVID-19 images. Second Data set: This dataset consists of 219 COVID-19 positive images and 1,341 negative COVID-19 images | Feature extraction using FrMEMs. Image classification using KNN | KNN image classification accuracy rates of 96.09% and 98.09% for the first and second datasets respectively |
| Hurt et al, (2020) [114] | To develop DL based diagnostic interpretation of COVID-19 from chest X-Ray. | X-Ray | 10 frontal chest radiographs from 5 patients with COVID-19. For training deep learning model 22K radiologist annotated radiographs | DL U-NET | Not reported |
| Joshi et al, (2020) [115] | To predict SARS-CoV-2 PCR positivity based on complete blood count components and patient sex. | Non-Imaging; (Blood count components and patient sex) | 33 confirmed SARS-CoV-2 PCR positive and 357 negative patients at Stanford Health Care were used for model training. Validation was done on multiple sets. Validation cohorts consisted of emergency department patients > 18 years old who had concurrent complete blood counts and SARS-CoV-2 PCR testing 1. Northern California (41 PCR positive, 495 PCR negative),  2. Seattle, Washington (40 PCR positive, 306 PCR negative),  3. Chicago, Illinois (245 PCR positive, 1015 PCR negative), and  4. South Korea (9 PCR positive, 236 PCR negative) | Logistic Regression | 1. Northern California: AUC=0.78  2. Seattle, Washington: AUC=0.75  3. Chicago, Illinois: AUC=0.76 4. South Korea: AUC=0.81 |
| Kang et al, (2020) [116] | Study proposes to conduct the diagnosis of COVID-19 with a series of features extracted from CT images. | CT | 2522 CT images involved in this study, where 1495 cases are from COVID-19 patients and the rest 1027 cases are from community-acquired pneumonia (CAP) patients | Multiple Models, Neural Network performed best | Standardized Neural Network Accuracy 93.90 ± 2.04 |
| Khan et al, (2020) [117] | To detect COVID-19 cases using chest X-Ray. | X-Ray | 1203 normal, 660 bacterial Pneumonia and 931 viral pneumonia cases. Study collected a total of 1300 images | CoroNet (Deep CNN based on Xception architecture) | Overall accuracy of 89.6% for 3–class classification (normal vs pneumonia vs COVID-19) |
| Khuzani et al, (2020) [118] | To reliably distinguish the X-Ray images of COVID-19 patients from other forms of pneumonia using machine learning-based classifiers. | X-Ray | 420 2-D X-Ray images, in the Posteroanterior (P.A.) chest view. Normal (140 images), pneumonia (140 images), and COVID-19 (140 images) | Multi-layer neural network with 2 hidden layers | COVID-19 detection precision 96% , Sensitivity 100% |
| Ko et al, (2020) [119] | To rapidly develop an AI technique to diagnose COVID-19 pneumonia in CT images and differentiate it from non–COVID-19 pneumonia and non-pneumonia diseases. | CT | 3993 chest CT images of patients with COVID-19 pneumonia, other pneumonia, and non-pneumonia diseases | Fast-track COVID-19 classification network (FCONet) | Sensitivity 99.58%, Specificity 100.00%, and Accuracy 99.87% |
| Li et al, (2020) [120] | To develop a fully automatic framework to detect COVID-19 using chest CT and evaluate its performances. | CT | 4,356 Chest exams from 3,322 patients | COVNET (ResNet50 as a backbone) | Identify COVID-19 on chest CT exams, AUC = 0.96. Identify community acquired pneumonia on chest CT exams AUC = 0.95 |
| Mei et al, (2020) [121] | To design an AI model that can identify SARS-CoV-2 infection based on initial chest CT scans and associated clinical information that could rapidly identify COVID-19 (+) patients in the early stage. | CT | 905 patients | Joint Model. CNN + MLP | AUC = 0.92 |
| Murphy et al, (2020) [122] | To evaluate the performance of an artificial intelligence (AI) system for detection of COVID-19 pneumonia on chest radiographs. | X-Ray | An AI system (CAD4COVID-Xray) was trained on 24,678 CXR images including 1,540 used only for validation while training. The test set consisted of a set of continuously acquired CXR images (n=454) | CAD4COVID-XRay (software developed by Thirona (Nijmegen, Netherlands)) | AUC of 0.81 |
| Oh et al, (2020) [123] | To build a COVID-19 diagnostic tool using patch-based convolutional neural network approach with relatively small number of trainable parameters. | X-Ray | Total curated data set = 502 radiography images (Including 180 COVID-19 images). For COVID-Net comparison used dataset from that study, 13,975 CXR images across 13,870 patient cases | Neural Network (ResNet-18 as a backbone) | Overall accuracy of 91.9 % against COVID-Net dataset comparison |
| Panwar et al, (2020) [124] | Develop a deep learning neural network-based method for detecting the COVID-19 by analyzing the X-Ray of patients. | X-Ray | 192 X-Ray images of COVID-19 positive patients and total 337 images, Kaggle’s Chest X-Ray images (pneumonia) consisting of 5863 images | Deep learning neural network-based method nCOVnet | AUC of ROC: 0.88095 |
| Pu et al, (2020) [125] | To define the uniqueness of chest CT infiltrative features associated with COVID-19 image characteristics as potential diagnostic biomarkers. | CT | Chest CT exams including n = 498 of 151 unique patients RT-PCR positive for COVID-19 and n = 497 unique patients with community-acquired pneumonia (CAP) | Convolutional neural networks (CNN) | AUC of 0.70 with 99% CI 0.56–0.85 |
| Rahimzadeh et al, (2020) [126] | To classify the chest X-Ray images into three categories of normal, pneumonia, and COVID-19 using Xception and ResNet50V2 neural network. | X-Ray | Two open-source datasets that contained 180 COVID-19 images, 6054 images from patients infected with pneumonia, and 8851 images from normal cases | Concatenated neural network based on Xception and ResNet50V2 | Overall accuracy of all classes is 91.4%. Accuracy for detecting COVID-19 cases is 99.50% |
| Rajaraman et al, (2020) [127] | To improve COVID-19 detection in X-Ray, with the baseline being the training data without augmentation. | X-Ray | Multiple publicly available CXR collections.  1) Pediatric CXR dataset  2) RSNA CXR dataset  3) CheXpert CXR dataset  4) NIH CXR‐14 dataset  5) Twitter COVID‐19 CXR dataset  6) Montreal COVID‐19 CXR dataset. For details refer to paper | Multiple Models evaluated including custom WRN model, VGG-16 had better accuracy | VGG-16 Accuracy: 0.9308 |
| Roy et al, (2020) [128] | To detect COVID-19 from lung ultrasonography (LUS) images. | Lung Ultrasound | 277 LUS from 35 patients | Deep network, derived from Spatial Transformer Networks. CNN+Reg-STN+SORD | Average F1-Score is 71.4 |
| Saiz et al, (2020) [129] | To develop a quick method to detect COVID-19 in chest X-Ray images using deep learning techniques. | X-Ray | 1500 images (non-infected patients, infected with COVID-19, and pneumonia) | Deep convolutional neural network (Feature extraction done using VGG-16) | 94.92% of sensibility and 92.00% of specificity in COVID-19 detection |
| Singh et al, (2020) [130] | To classify the COVID-19-infected patients as infected (positive) or not (Negative). | CT | Not clear | Multi-objective differential evolution (MODE)-based Convolutional neural networks (CNN) | Not clear |
| Singh et al, (2020) [99] | To build a deep learning model for detection of COVID-19 from chest images. | X-Ray | The dataset consists of 1419 images comprising 132 images of COVID cases, 619 cases of viral pneumonia and 668 healthy cases | Modified XceptionNet | Detection of COVID-19 Accuracy is reported as 95.80% |
| Song et al, (2020) [100] | To identify COVID-19 pneumonia patients in real time using CT. | CT | A total of 201 patients that includes 98 patients with confirmed COVID-19 pneumonia (positive), and 103 patients with confirmed non-COVID-19 pneumonia (negative)) | Semantic features from the CT images extracted by bi-directional generative adversarial network (BigBiGAN). Linear classifier used as the end model | AUC: 0.972 |
| Togacar et al, (2020) [101] | To differentiate between coronavirus, pneumonia, and normal using deep learning. | X-Ray | Total 458 Images. 295 images in COVID-19 class. Normal class X-Ray images are 65 in total, and pneumonia class X-Ray images are 98 | Support Vector Machines as the end model | Overall classification rate was 99.27% |
| Ozturk et al, (2020) [102] | To develop a model for automatic COVID-19 detection using raw chest X-Ray images. | X-Ray | Data used from two sources.  Source 1: 127 COVID-19 X-Ray images. Source 2: 500 no-findings and 500 pneumonia class frontal chest X-Ray images from literature | DarkCovidNet for classification. (Built on top of deep neural network model Darknet-19) | Accuracy of 98.08% for binary classes and Accuracy of 87.02% for multi-class cases |
| Tuncer et al, (2020) [103] | To automatically detect COVID-19. | X-Ray | Total of 234 healthy X-Ray chest images | SVM | SVM classifier achieved 100.0% classification accuracy by using 10-fold cross-validation |
| Ucar et al, (2020) [104] | To develop an intelligent diagnosis system for COVID-19. | X-Ray | 5949 posteroanterior chest radiography images for 2839 patient cases from COVIDx database. The dataset includes 1583 normal, 4290 pneumonia and 76 COVID-19 infection cases | COVIDiagnosis-Net, based on deep SqueezeNet with Bayes optimization | Accuracy in diagnosing COVID-19 was 0.983 |
| Vaid et al, (2020) [105] | To precisely predict COVID-19 positive cases from chest X-Ray scans. | X-Ray | Chest X-Ray images from 181 patients | Deep learning model relying on convolutional neural networks | Accuracy in diagnosing COVID-19 was 96.3% |
| Waheed et al, (2020) [106] | To generate synthetic chest X-Ray (CXR) images and demonstrate that the synthetic images produced from CovidGAN and can be utilized to enhance the performance of COVID-19. | X-Ray | 1124 CXR images. More precisely, there are 403 images of COVID-CXR and 721 images of normal-CXR | CNN-AD CNN-SA | COVID-19 detection accuracy. CNN-AD: Accuracy 85% CNN-SA: Accuracy 95% |
| Warman et al, (2020) [107] | To effectively diagnose COVID-19 using artificial intelligence, instead of or in addition to reverse transcription-polymerase chain reaction (RT-PCR), can improve widespread COVID-19 detection and resource allocation. | CT | 904 axial lung window CT slices from 338 patients in 17 countries. This includes 606 images from COVID-19 positive patients, 224 images of variety of other pulmonary diseases, and 74 images of normal patients | Deep learning model combined with decision tree model | On an independent test of 219 images from COVID-19 positive, a variety of pneumonia, and healthy patients, the model predicted COVID-19 diagnoses with an accuracy of 96.80 %. On an independent test of 34 images from asymptomatic COVID-19 positive patients, model achieved an accuracy of 97.06% |
| Wu et al, (2020) [108] | To develop a deep learning-based method to assist radiologists to fast and accurately identify patients with COVID-19 by CT images. | CT | CT images of 495 patients from three hospitals in China | Multi-view deep learning fusion (Based on ResNet50 architecture) | AUC of 0.819 |
| Xie et al, (2020) [109] | Study proposes a relational approach (RTSU-Net) that leverages structured relationships by introducing a novel non-local neural network module. | CT | RTSU-Net on a cohort of 5000 subjects from the COPDGene study (4000 for training and 1000 for evaluation) Transfer learning to retrain and evaluate RTSU-Net on 470 COVID-19 suspects (370 for retraining and 100 for evaluation) | RTSU-Net | Refer to paper for Qualitative and Quantitative performance results |
| Xu et al, (2020) [110] | To develop a federated learning-based unified CT-COVID AI Diagnostic tool. | CT | 5732 CT images from 1276 individuals | CNN | CNN achieved a sensitivity of 97.5% and specificity of 89.4% in differentiating COVID-19 from three types of non-COVID-19 cases |
| Yang et al, (2020) [111] | To evaluate the diagnostic efficacy of Densely Connected Convolutional Networks (DenseNet) for detection of COVID-19 features on high resolution computed tomography (HRCT). | CT | 295 (healthy person: 149; COVID-19 patients: 146) | Neural network: DenseNet | DenseNet AUC 0.98 (95% CI: 0.972–0.995) in the test set |
| Yi et al, (2020) [112] | Study aim to re-purpose the algorithms built and trained to identify other pneumonias for detecting COVID-19. | X-Ray | 88 frontal (chest radiographs) CXRs with confirmed COVID-19 diagnoses from 3 radiology repositories | Deep convolutional neural network | AUROC: 0.87 to 0.91 |

List of Abbreviation used in Multimedia Appendix 3

| **Abbreviation** | **Description** |
| --- | --- |
| AI | Artificial Intelligence |
| AUC | Area Under the Curve |
| AUROC | Area Under the Receiver Operating Characteristics |
| BigBiGAN | Big bidirectional Generative Adversarial Network |
| CAP | Community Acquired Pneumonia |
| CNN | Convolutional Neural Network |
| CNN-AD | CNN used on actual data |
| CNN-SA | CNN used on synthetic images |
| CT | Computed Tomography |
| CXR | Chest X-Ray |
| DI2IN | Deep Image-to-Image Network |
| DL | Deep Learning |
| FrMEMs | Fractional Multichannel Exponent Moments |
| KNN | K-Nearest Neighbors |
| LHOS | Lung High Opacity Score |
| LSS | Lung Severity Score |
| LUS | Lung Ultrasound |
| ML | Machine Learning |
| MLP | Multilayer Perceptron |
| NIH | National Institutes of Health |
| PCR | Polymerase Chain Reaction |
| PHO | Percentage of High Opacity |
| PO | Percentage of Opacity |
| ROC | Receiver Operating Characteristic |
| RSNA | Radiological Society of North America |
| SORD | Soft ORDinal regression |
| STN | Spatial Transformers Network |
| SVM | Support Vector Machine |
