# Supplementary material for: Role of Machine Learning Techniques to Tackle the COVID-19 Crisis: Systematic Review
Source: JMIR Med Inform. 2021 Jan 11;9(1):e23811. doi: 10.2196/23811 (PMC7806275; doi:10.2196/23811)
Supplement: Multimedia Appendix 4 [file medinform_v9i1e23811_app4.docx]

Appendix 4: Details of 19 studies qualified under the Disease Progression (DP) theme.

| **Study** | **Aim** | **Category** | **Dataset Variables** | **Data Source** | **Date Range** | **Sample Size** | **ML Model** | **ML Model Performance** |
| --- | --- | --- | --- | --- | --- | --- | --- | --- |
|  |  |  |  |  |  |  |  |  |
| Al-Najjar et al, (2020) [131] | To build a classifier that predicts status of recovered and death of confirmed coronavirus patients in South Korea. | Risk Stratification | 7 variables : country, infection reason, sex, group, confirmation date, birth year, and region | Data from Korea Centers for Disease Control and Prevention (KCDC). Region: South Korea | Feb 20 2020 - March 9 2020 | 7,869 coronavirus patients | A neural network with one hidden layer and gradient descent as an optimization algorithm | Patient recovery accuracy 93.8%. Patient mortality accuracy 99.5% |
| Burian et al, (2020) [132] | To evaluate clinical and imaging parameters for estimating the need of intensive care unit (ICU) treatment and machine learning modelling to estimate the risk for ICU treatment. | Hospital Resource Management | Demographics (age, sex), comorbidities, medical history, physical examination, lab results (complete blood count, leukocytes, lymphocytes, creatinine, creatine kinase, creatine kinase MB, CRP, troponine T, LDH, D-dimer, IL-6 ), and radiological variables | Data from Technical University of Munich. Region: Germany | March and April 2020 | 65 COVID-19 patients | Random Forest | 5-fold cross validation of Random Forest model: ROC-AUC of 0.79 ± 0.1 |
| Chan et al, (2020) [133] | To provide information about frequency, outcomes and recovery associated with AKI and dialysis in hospitalized COVID-19 patients. | Risk Stratification | Demographics (age, sex, race, ethnicity), vital signs, diagnosis, comorbidities, lab results, and other clinical variables | Data from Mount Sinai Health System (MSHS), New York. Region: United States | February 27 and April 15, 2020 | 3,235 hospitalized patients; Age>=18 | XGBoost | AUC 0.79 |
| Cheng et al, (2020) [134] | To explore whether quantitative computer tomography (CT) could be used to assess severity on admission. | Risk Stratification | Demographics (age, sex), comorbidities, vital signs, lab results (WBC, BUN etc.), radiological variables | All participants in the study had direct or indirect contact history with people from Wuhan. Region: China | January 17 to February 9, 2020 | 38 hospitalized patients with COVID-19 | Multi-scale convolutional neural networks for segmentation | Not Applicable |
| Cheng et al, (2020) [135] | To build a machine learning-based risk prioritization tool that predicts ICU transfer within 24 hours. | Hospital Resource Management | Demographics (age, sex, race, ethnicity) , vital signs, nursing assessments, lab results ( C-reactive protein, White blood cell count, Lymphocyte count etc.), and electrocardiograms | Data from Mount Sinai Health System. Region: United States | 26 February and 18 April 2020 | Overall 1987 unique patient; Test set of 612 patients | Random Forest | AUROC = 79.9% |
| Du et al, (2020) [136] | To evaluate lung lesion CT radiological features along with quantitative analysis for the COVID-19 patients that are ready for discharge. | Hospital Resource Management | Demographics (age, sex), CT, RT-PCR | Data from Fangcang shelter hospital in Hongshan Gymnasium, Wuhan. Region: China | February 10 to March 10, 2020 | 125 COVID-19 patients | InferRead™ CT Pneumonia software (Beijing Infervision Technology Co., Ltd., Beijing, China) | Not Applicable |
| Fu et al, (2020) [16] | To use the radiomics signatures of the whole lung as a machine learning-based tool to evaluate the prognosis of patients with coronavirus disease. | Risk Stratification | Demographics (age, sex), vital signs, lab results (White blood cell count, Lymphocyte count, C-reactive protein, Liver function, Myocardial enzymes), other clinical variables, and CT images | Data from Chinese Health Commission. Region: China | January 21 to February 19, 2020 | 64 confirmed COVID-19 patients | SVM Classifier | AUC=0.833 |
| Ji et al, (2020) [137] | To build a predictive model for identifying high-risk non-severe pneumonia patients at early stage. | Risk Stratification | Demographics (age, gender), vital signs, comorbidities, lab results, other clinical variables, radiological variables from CT images | Data from Renmin Hospital of Wuhan University. Region: China | January 2nd to 20th, 2020 January 28th to February 9th, 2020 | 148 patients | Logistic regression | 0.759 (95% CI: 0.635–0.884) |
| Jiang, et al, (2020) [138] | To develop a tool with AI capabilities that will predict patients at risk for more severe illness on initial presentation. | Risk Stratification | Demographics (age, sex), vital signs, comorbidities, lab results (white blood cell, Hemoglobin etc.), radiological variables, and other clinical variables | Data from two hospitals in Wenzhou, Zhejiang, China. Region: China | Not Available | 53 hospitalized patients with COVID-19 | Multiple Models KNN and SVM outperformed | KNN=80% Accuracy in predicting severe cases. SVM=80% Accuracy in predicting severe cases |
| Li et al, (2020) [139] | To develop an automated measure of COVID-19 pulmonary disease severity on chest radiographs (CXRs), for longitudinal disease evaluation and clinical risk stratification. | Risk Stratification | Demographics (age, gender), clinical and radiological variables | Data from Stanford Hospital and from other hospitals including Massachusetts General Hospital. Region: United States | March -April, 2020 | ~160,000 images from Stanford Hospital, Palo Alto. 314 chest X-Ray from patients with COVID-19 (Massachusetts General Hospital, Boston, MA). Evaluated on internal and external test sets from different hospitals, containing 154 and 113 X-Ray respectively | Convolutional siamese neural network model | AUROC=0.80 |
| McRae et al, (2020) [140] | To customize point-of-care diagnostic tool that is suitable for the measurement of biomarkers that can be used to discriminate between COVID-19 patients that recover vs. those that die from complications. | Risk Stratification | Demographics (age, sex), comorbidities, and measurements of C-reactive protein (CRP), N-terminus pro B type natriuretic peptide (NT-proBNP), myoglobin (MYO), D-dimer, procalcitonin (PCT), creatine kinase-myocardial band (CK-MB), and cardiac troponin I (cTnI) | Data from Wuhan and Shenzhen in China. Region: China | 160 Wuhan January 23, 2020, to February 23, 2020 12 patients from Shenzhen as of Jan 21 2020 | 160 hospitalized COVID-19 patients | Logistic regression | COVID-19 Severity Score. AUC 0.94 |
| Shashikumar et al, (2020) [141] | To develop, externally validate and prospectively test a transparent deep learning algorithm for predicting 24 hours in advance the need for mechanical ventilation in hospitalized patients and those with COVID-19. | Hospital Resource Management | Demographics (age, sex, race), vital signs (heart rate, oxygen saturation, respiratory rate, FiO2, and pH), lab results (Blood urea nitrogen, Alkaline phosphate, Bilirubin direct etc.) ,SOFA and CCI scores | Data from University of California, San Diego Health (UCSD), and the Massachusetts General Hospital (MGH). Region: United States | Two academic medical centers from January 01, 2016 to December 31, 2019 (Retrospective cohorts) and February 10, 2020 to May 4, 2020 (Prospective cohorts) | Over 31,000 admissions to the intensive care units (ICUs) at two hospitals. Additionally, 777 patients with COVID-19 were used for prospective validation | VentNet (a two layer feedforward neural network) | The performance of the model with a 24-hour prediction horizon at validation is: AUC=0.882 for general ICU population. AUC=0.918 for patients with COVID-19 |
| Shen et al, (2020) [142] | Study hypothesize that SARS-CoV-2 induces characteristic molecular changes that can be detected in the sera of severe patients. | Risk Stratification | Demographics (age, sex), social history (BMI, smoke, alcohol), radiology, clinical variables, comorbidities, vital signs, medications, proteomic and metabolomics profiling of sera | Data from Zhejiang Province. Region: China | January 2020 through March 2020 | 46 COVID-19 and 53 control individuals for training. 19 additional COVID-19 patients from second test cohort | Random Forest | Identification of Severe patients using Random Forest on training set with an AUC: 0.957 |
| Wang et al, (2020) [143] | To develop a quantitative method based on deep-learning (DL) segmentation to check the ARDS status in COVID-19 patients during treatment. | Risk Stratification | Radiological variables from chest CT | Training cases were from Shanghai, Jiangsu and Wuhan Province of China. Region: China | Jan to Feb 2020 COVID-19 patients | Trained by 2,565 COVID-19 cases, and 2,785 negative cases | VB-Net model for segmentation | Not Applicable |
| Wang et al, (2020) [144] | To determine the patterns of chest computed tomography (CT) evolution according to disease severity in a large coronavirus disease. | Risk Stratification | Demographics (age, sex), lab results (arterial oxygen saturation, blood cell count, biomarkers of inflammation, hepatic and renal function and coagulation), comorbidities, and radiological variables | Data from Jiangsu Province, China. Region: China | January 10, 2020, to February 18, 2020 | 484 patients | DL model ( Deepwise & League of PhD Technology Co., Ltd) was used for lung segmentation and lesion extraction | Not clear |
| Wang et al, (2020) [145] | To build COVID-19 diagnostic and prognostic model using routinely used computed tomography. Classify patients in to high-risk and low-risk groups whose hospital-stay time have significant difference. | Risk Stratification | Demographics (age, sex), comorbidities, pneumonia (from COVID-19, Bacterial, Mycoplasma, Viral, and Fungal), CT Images | Data from 7 cities or provinces of China including Wuhan. Region: China | 1) Pneumonia CTs before Dec. 2019. 2) COVID-19 pneumonia CTs from Dec 2019 | Total of 5,372 patient’s data collected. 4106 patients with computed tomography images were used to pre-train the DL system. 1266 patients (924 with COVID-19, and 471 had follow-up for 5+ days; 342 with other pneumonia) for testing and validation | COVID-19Net (DenseNet121-FPN for lung segmentation ) | COVID-19Net achieved good performance in identifying COVID-19 from other pneumonia (AUC=0.87 and 0.88) and viral pneumonia (AUC=0.86) |
| Wollenstein-Betech et al,  (2020) [148] | Apply ML model to develop personalized models to predict hospitalization, mortality, need for ICU, and need for a ventilator. | Risk Stratification | Demographics (age, gender), chronic renal insufficiency, diabetes, immunosuppression, pregnancy, cardiovascular disease, asthma, obesity, and SARS-CoV-2 test result. | Open source dataset made available by Mexico government. | Until May 1^st^ 2020. | ~91,000 patients | Logistic Regression, SVM,  Random Forest, and XGBoost | Accuracy of predicting hospitalizations: SVM, Logistic Regression : 0.609  Accuracy of predicting mortality: Logistic Regression: 0.729.  Refer to paper for detailed accuracy. |
| Yadaw et al, (2020) [146] | To accurately predict mortality among COVID-19 patients. | Risk Stratification | Age, Sex, Race, Encounter type, Temperature, Systolic BP, Diastolic BP, O2_SAT, O2SAT_min, Smoking, Comorbidities (Asthma, COPD, Hypertension, Obesity, Diabetes, HIV, Cancer), Tociluzumab, Hydroxychloroquine, Azithromycin | Data from Mount Sinai Health System in New York. Region: United States | Data collected through April 7, 2020 | 5,051 COVID-19 patients | XGBoost | AUC of best performing XGBoost model. Using 5 features Test set 1: 0.91 Using 5 features Test set 2: 0.91 Using 20 features Test set 1: 0.91 Using 20 features Test set 2: 0.95 |
| Zhang et al, (2020) [147] | To build an AI system that can diagnose COVID-19 pneumonia using CT scans and progression of disease to critical illness. | Risk Stratification | Demographics (age, sex), vital signs (temperature, oxygen saturation etc.), lab results (Hemoglobin, White Blood cell, Albumin, Platelet etc.), and radiological variables | Data from China Consortium of Chest CT Image Investigation (CC-CCII). Region: China | Multiple date ranges by type of data variables. Refer paper for details | 3,777 patients (532,506 CT slices) | DeepLabv3 for CT images segmentation. LightGBM for progression to critical illness | DeepLabv3 (mDC: 0.587±0.012 and mPA: 0.662±0.011). Prediction of progression to critical illness AUROC = 0.9093 (95% CI: 0.8775–0.9369) |

List of Abbreviation used in Multimedia Appendix 4

| **Abbreviation** | **Description** |
| --- | --- |
| AI | Artificial Intelligence |
| AKI | Acute Kidney Injury |
| ARDS | Acute Respiratory Distress Syndrome |
| AUC | Area Under the Curve |
| AUROC | Area Under the Receiver Operating Characteristics |
| BMI | Body Mass Index |
| BP | Blood Pressure |
| BUN | Blood Urea Nitrogen |
| CCI | Charlson Comorbidity Index |
| CI | Confidence Interval |
| COPD | Chronic Obstructive Pulmonary Disease |
| CRP | C-Reactive Protein |
| CT | Computed Tomography |
| CXR | Chest X-Ray |
| DL | Deep Learning |
| FiO2 | Fraction of Inspired Oxygen |
| HIV | Human Immunodeficiency Viruses |
| ICU | Intensive Care Unit |
| KNN | K-Nearest Neighbors |
| LDH | Lactate Dehydrogenase |
| LightGBM | Light Gradient Boosting Machine |
| MA | Massachusetts |
| MB | Myocardial Band |
| mDC | mean Dice Coefficient |
| ML | Machine Learning |
| mPA | mean Pixel Accuracy |
| O2 | Oxygen |
| O2SAT | Oxygen saturation |
| PhD | Doctor of Philosophy |
| ROC-AUC | Receiver Operating Characteristics - Area Under the Curve |
| RT-PCR | Reverse Transcription Polymerase Chain Reaction |
| SARS-Cov | Severe acute respiratory syndrome coronavirus |
| SAT | Saturation |
| SOFA | Sequential Organ Failure Assessment |
| SVM | Support Vector Machine |
| WBC | White Blood Cell |
